# C-951-04. Evaluating the Impact of Burn Extent on Antibiotic Prescribing and Clinical Outcomes

**DOI:** 10.1093/jbcr/irag033.112

**Published:** 2026-04-06

**Authors:** Esha Kelkar, Juquan Song, Amina El Ayadi, Steven E Wolf

**Affiliations:** University of Texas Medical Branch, Galveston, TX; University of Texas Medical Branch, Galveston, TX; Division of Burn and Trauma Surgery, Department of Surgery, University of Texas Medical Branch, Galveston, TX; Division of Burn and Trauma Surgery, Department of Surgery, University of Texas Medical Branch, Galveston, TX

## Abstract

**Introduction:**

Antibiotic prophylaxis regimens in burn patients lack consistency and are often individualized by patient presentation. While antibiotic prophylaxis offers numerous benefits, its overuse can lead to antibiotic resistance, adverse drug reactions, and disruption of the body’s microbiome, ultimately making infections harder to treat. The purpose of this study is to investigate clinical outcomes in burn patients receiving antibiotics by percent of total body surface area burned to determine whether burn size should determine antibiotic prescribing patterns.

**Methods:**

Patients with burn injuries who were given penicillins or beta-lactam antimicrobials within 7 days of their injury were identified on the TriNetX database. The patient population was stratified by percent of total body surface area (% TBSA) burned in the intervals of <10%, 10-19%, 20-29%, 30-39%, and ≥ 40% TBSA. The cohorts were balanced using propensity score matching of patient demographic factors, burn/corrosion, and pre-existing conditions, including diabetes mellitus, acute kidney failure, chronic kidney disease, and immunodeficiencies. Among the ten balanced cohorts, the sample size varied from 1033 to 36 271 patients. Outcomes analyzed were local skin infections, sepsis, acute kidney failure, mortality, and pneumonia within 7 days and 30 days of the injury.

**Results:**

Antibiotic use is associated with a significantly increased risk for all chosen outcomes within both 7 days and 30 days for minor burns (< 10% TBSA, 10-19% TBSA). The association with adverse outcomes is strongest in patients with minor burns, and becomes weaker as burn size increases. Outcomes such as sepsis and mortality demonstrate the highest risk ratios in patients with the smallest burns, while large burns (≥40% TBSA) show a less significant risk difference between antibiotic and non-antibiotic groups. Notably, this pattern is inconsistent in moderate-sized burns (20-29% TBSA, 30-39% TBSA).

**Conclusions:**

Antibiotic use in burn patients, especially those with minor to moderate burns, is associated with a significantly higher risk of adverse outcomes at both 7 and 30 days post-injury. This suggests that routine antibiotic use in minor burns may be harmful; its benefit in severe burns is less clear and may not outweigh the risks.

**Applicability of Research to Practice:**

Clinicians may choose to withhold antibiotic prescription in patients with minor burns due to its association with poorer outcomes.

**Funding for the study:**

N/A.

